# Molecular Mechanisms of Palmitic Acid Augmentation in COVID-19 Pathologies

**DOI:** 10.3390/ijms22137127

**Published:** 2021-07-01

**Authors:** Christie Joshi, Viren Jadeja, Heping Zhou

**Affiliations:** Department of Biological Sciences, Seton Hall University, South Orange, NJ 07079, USA; christie.joshi@student.shu.edu (C.J.); viren.jadeja@student.shu.edu (V.J.)

**Keywords:** obesity, palmitic acid, n-3 fatty acid, COVID-19, cytokines, chemokines

## Abstract

The coronavirus disease 2019 (COVID-19) pandemic has claimed over 2.7 million lives globally. Obesity has been associated with increased severity and mortality of COVID-19. However, the molecular mechanisms by which obesity exacerbates COVID-19 pathologies are not well-defined. The levels of free fatty acids (FFAs) are elevated in obese subjects. This study was therefore designed to examine how excess levels of different FFAs may affect the progression of COVID-19. Biological molecules associated with palmitic acid (PA) and COVID-19 were retrieved from QIAGEN Knowledge Base, and Ingenuity Pathway Analysis tools were used to analyze these datasets and explore the potential pathways affected by different FFAs. Our study found that one of the top 10 canonical pathways affected by PA was the coronavirus pathogenesis pathway, mediated by key inflammatory mediators, including PTGS2; cytokines, including IL1β and IL6; chemokines, including CCL2 and CCL5; transcription factors, including NFκB; translation regulators, including EEF1A1; and apoptotic mediators, including BAX. In contrast, n-3 fatty acids may attenuate PA’s activation of the coronavirus pathogenesis pathway by inhibiting the activity of such mediators as IL1β, CCL2, PTGS2, and BAX. Furthermore, PA may modulate the expression of ACE2, the main cell surface receptor for the SARS-CoV-2 spike protein.

## 1. Introduction

The coronavirus disease 2019 (COVID-19) is caused by infection of severe acute respiratory syndrome coronavirus 2 (SARS-CoV-2). To date, there have been more than 120 million confirmed COVID-19 cases and more than 2.7 million deaths reported by the World Health Organization (WHO) [1]. While some may be asymptomatic, many COVID-19 patients exhibit symptoms such as fever, dry cough, shortness of breath, and fatigue [2]. Critically ill patients may experience cytokine storm and macrophage activation syndrome with profound inflammation and multi-organ dysfunction [3,4]. The risk factors for severe illness include age and comorbidities such as cardiovascular disease, diabetes, chronic respiratory disease, and cancer [5]. For example, the mortality rate of COVID-19 is increased to 14.8% in patients above 80 years old, 10.5% in those with pre-existing cardiovascular diseases, and 7.3% in those with diabetes [6]. Progression to severe illness is more frequent in obese patients than the normal weight group [7]. Simonnet et al. reported that 47.6% of severe cases have a body mass index (BMI) greater than 30 kg/m^2^ and the need for interventional mechanical ventilation increases with BMI independent of age, diabetes, and hypertension [8]. Consistently, Gao et al. reported a nearly linear relationship between BMI and the severity of COVID-19 [9].

Angiotensin converting enzyme 2 (ACE2) has been reported as the main cell surface receptor involved in host cell entry of SARS-CoV-2 [10]. It is expressed in more than 150 different cell types corresponding to all major human tissues and organs including the central nervous system (CNS) [10,11]. In the course of COVID-19 progression, systemic hyper-inflammation may lead to the development of cytokine storm involving many different cytokines, including interleukin (IL)6, IL8, tumor necrosis factor (TNF)α, and interferon (IFN)γ [12,13]. Elevation in the level of TNF, a key pro-inflammatory cytokine, is reportedly associated with increased COVID-19 mortality [14]. The circulating levels of IL-8 and IL-6 are significantly higher in COVID-19 patients admitted to an intensive care unit (ICU) or who died [15,16]. The circulating level of IFNγ is higher in both severe and mild COVID-19 patients while transforming growth factor (TGF)β in severe COVID-19 patients is significantly higher than that in mild COVID-19 patients and negative controls [17]. Furthermore, there is also evidence suggesting the involvement of Nod-like receptor family pyrin domain-containing 3 (NLRP3) inflammasome activation in the immuno-pathogenesis of severe COVID-19 by driving the cleavage of caspase-1 and secretion of IL1β and IL18 and other damage-associated molecular patterns (DAMPs) [18,19]. 

Obesity is an increasingly prevalent global health issue. According to the report by the WHO in 2016, more than 1.9 billion adults are overweight, and more than 650 million of those are obese [20]. In the United States, 71.6% of adults aged 20 and older are overweight, including 39.8% being obese based on the data from National Center for Health Statistics in 2015 to 2016 [21]. Chronic dyslipidemia is commonly observed in obese patients [22] and elevated levels of free fatty acids (FFAs) are believed to be a causal element of several obesogenic pathologies including diabetes, cardiovascular diseases [23,24], and nonalcoholic fatty liver disease (NAFLD) [25]. Furthermore, long chain saturated fatty acids (SFAs) such as palmitic acid (PA) have been reported to induce cellular dysfunction and lipotoxicity in a plurality of non-adipose tissues while unsaturated fatty acids exhibit low lipotoxicity or are often antagonistic of the deleterious effects from SFAs [26,27,28]. Long-chain SFAs have also been reported to induce the expression of inflammatory markers in various cells and tissues [29], while significant evidence shows that n-3 fatty acids exhibit anti-inflammatory effects in immune cells [30,31,32,33]. 

Obesity-associated chronic low-grade inflammation and immune dysregulation [29] may contribute to more severe outcomes in obese COVID-19 patients. However, the molecular mechanisms by which obesity augments COVID-19 pathologies are not clearly defined. Therefore, this study used Ingenuity Pathway Analysis (IPA), a bioinformatics tool from QIAGEN, to examine the molecular mechanisms underlying the obesity augmentation of COVID-19 pathologies. Molecules affected by obesity and COVID-19 were obtained from QIAGEN Knowledge Base (QKB), a repository of manually curated biological information, and used for IPA Core Analysis. Differential impacts of PA and n-3 fatty acids on the coronavirus pathogenesis pathway were analyzed, and the modulation of angiotensin-converting enzyme 2 (ACE2), the main cell surface receptor for the SARS-CoV-2 spike protein, by PA was examined.

## 2. Results

### 2.1. Canonical Pathway Analysis of PA-Associated Molecules

**Figure 1:**
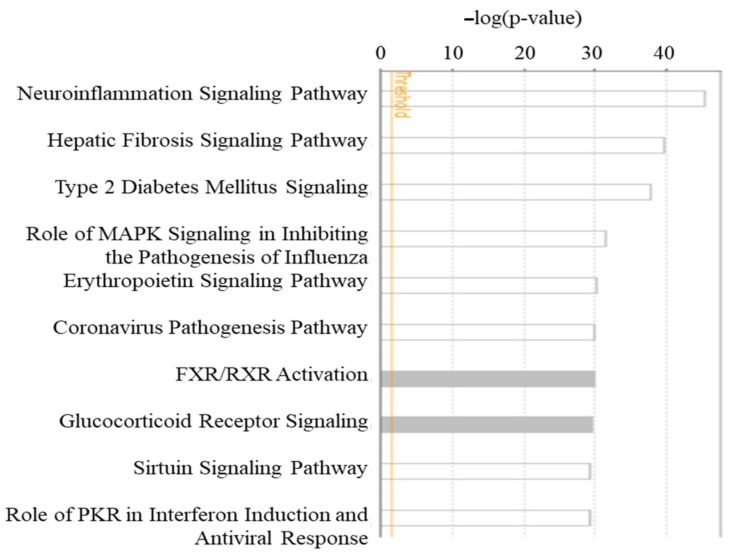
Canonical pathway analysis of PA-associated molecules. PA-associated molecules were retrieved from QKB and subjected to Core Analysis in IPA. Top 10 canonical pathways were presented with their respective *p*-values calculated using Benjamini–Hochberg corrected Fisher’s exact test.

Molecules associated with PA were retrieved from QKB using the IPA’s “Grow” tool. Chemicals were trimmed from the dataset, and 374 biological molecules were identified. These molecules were then analyzed using Core Analysis to identify canonical pathways associated with these molecules. As shown in Figure 1, the canonical pathway with the lowest *p* value was the neuroinflammation signaling pathway (*p* < 3.59 × 10^−46^). hepatic fibrosis signaling pathway (*p* < 2.48 × 10^−40^) and type 2 diabetes mellitus signaling pathway (*p* < 2.14 × 10^−38^) were also identified among the top 10 pathways, which is consistent with many studies reporting the roles of increased levels of SFAs, especially PA, in the development of liver fibrosis, NAFLD [34] and type 2 diabetes [35]. Moreover, our core analysis also identified three pathways, namely the role of MAPK signaling in inhibiting the pathogenesis of influenza (*p* < 3.86 × 10^−32^), coronavirus pathogenesis pathway (*p* < 1.79 × 10^−30^), and role of PKR in interferon induction and antiviral response (*p* < 4.98 × 10^−30^), all of which play key roles in antiviral response and pathogenesis following viral infections. These findings suggest that PA may have a significant impact on our body’s response to viral infections. In this study, we chose to focus on the coronavirus pathogenesis pathway. 

### 2.2. Overlapping of Molecules Associated with PA and Those Associated with COVID-19

**Figure 2:**
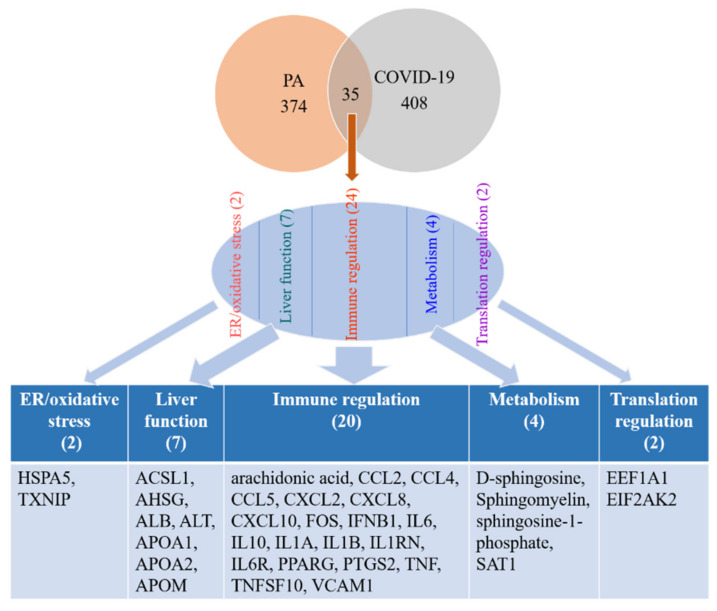
35 biological molecules were found to be overlapped between PA- and COVID-19-associated molecules, including those involved in ER/oxidative stress, liver function, immune regulation, metabolism, and translation regulation.

Molecules associated with COVID-19 were retrieved from QKB. Chemicals were trimmed from the dataset and 408 biological molecules were identified. These molecules were compared with 374 biological molecules associated with PA and 35 molecules were found to be overlapped between PA- and COVID-19-associated molecules (Figure 2). Among these 35 molecules, two molecules—heat shock protein family A Member 5 (HSPA5) and thioredoxin interacting protein (TXNIP)—were involved in endoplasmic reticulum (ER)/oxidative stress; two molecules—eukaryotic elongation factor 1-alpha 1 (EEF1A1) and eukaryotic translation initiation factor 2 alpha kinase 2 (EIF2AK2)—were involved in translation regulation; four molecules were involved in metabolism; seven molecules were involved in liver function; and 20 molecules were involved in immune regulation, including prostaglandin-endoperoxide synthase 2 (PTGS2), also known as cyclooxygenase-2 (COX-2), the key enzyme involved in prostaglandin biosynthesis from arachidonic acid (AA), transcription factor FOS, cytokines such as IL1β, IFNB1, and IL6, and chemokines such as C-C motif chemokine ligand 2 (CCL2), C-X-C motif chemokine ligand 8 (CXCL8), and C-C motif chemokine ligand 5 (CCL5) (Figure 2), which suggests that PA and COVID-19 may converge on their effects on ER/oxidative stress, liver function, metabolism, translation regulation, and immune regulation. 

### 2.3. Paths from PA to Coronavirus Pathogenesis Pathway

**Figure 3:**
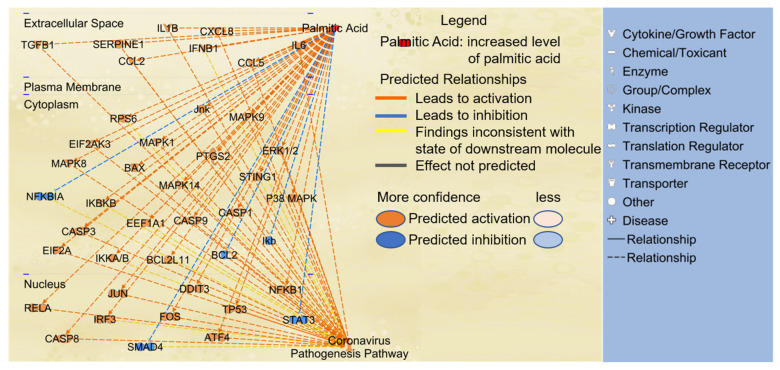
The paths from PA to coronavirus pathogenesis pathway were established using the “Path Explorer” tool in IPA, and increased level of PA was simulated using the “MAP” tool in IPA.

Next, we examined the potential paths by which PA may affect the coronavirus pathogenesis pathway. Using IPA’s “Path Explorer” tool, the 38 shortest paths from PA to coronavirus pathogenesis pathway were established and are shown in Figure 3. Some of the overlapping molecules between PA- and COVID-19-associated molecules were identified on the shortest paths, including FOS, EEF1A1, IL1B, IFNB1, IL6, CCL2, CXCL8, CCL5, and PTGS2 (Figure 3). Eukaryotic translation initiation factor 2-alpha kinase (EIF2AK)2 and EIF2AK3 encode kinases that phosphorylate EIF2A. Then, the “Molecule Activity Predictor (MAP)” tool was used to predict the effects of increased level of PA on the coronavirus pathogenesis pathway. The simulated increase in the level of PA enhanced the activity of transcription factors such as FOS, interferon regulatory factor 3 (IRF3), and NFκB; molecules involved in apoptosis, including BAX, caspase (CASP)3, CASP8, and CAPS9, translation regulatory molecules including EEF1A1; and inflammatory mediators including MAPKs, PTGS2, CASP1, cytokines, and chemokines. An increased level of PA was also predicted to decrease the activity of the inhibitor of nuclear factor kappa B1 (IκB1) and NFκB inhibitor alpha (NFκB1A), which inhibit the activity of NFκB; attenuate the activity of mothers against decapentaplegic homolog 4 (SMAD4), which suppresses IFNγ [36]; and diminish the activity of BCL2, which encodes an integral outer mitochondrial membrane protein that blocks the apoptotic death of some cells [37], thereby promoting inflammation and apoptosis. 

### 2.4. Effects of n-3 Fatty Acids on the Path from PA to Coronavirus Pathogenesis Pathway

**Figure 4:**
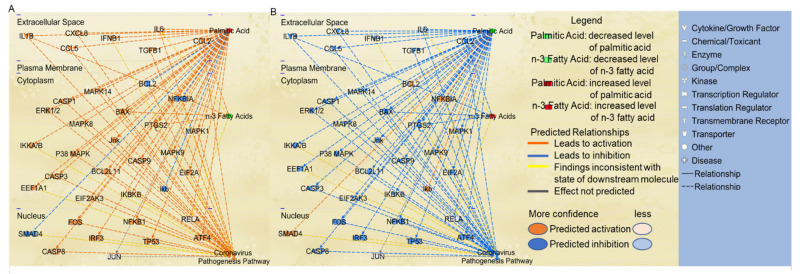
The effects of n-3 fatty acids on the paths from PA to coronavirus pathogenesis pathway using the “Path Explorer” and “MAP” tools in IPA. (**A**) Increased level of PA and decreased level of n-3 FAs were simulated using the “MAP” tool in IPA. (**B**) Decreased level of PA and increased level of n-3 FAs were simulated using the “MAP” tool in IPA.

Considering that n-3 fatty acids have been reported to exhibit anti-inflammatory activities, we used the “MAP” tool to predict the effects of n-3 fatty acids on the coronavirus pathogenesis pathway. A decreased level of n-3 fatty acids was predicted to augment the activity of BAX, IL1B, CCL2, and PTGS2 (Figure 4A). An increased level of PA and decreased level of n-3 fatty acids altogether significantly augmented the activation of coronavirus pathogenesis pathway (Figure 4A). In contrast, the increased level of n-3 fatty acids exhibited inhibitory effects on IL1B, CCL2, PTGS2, and BAX (Figure 4B). A decreased level of PA and increased level of n-3 fatty acids altogether reduced the activity of molecules involved in apoptosis including BAX, and decreased the activity of inflammatory mediators, including PTGS2, cytokines, including IL1B, and chemokines, including CCL2, thereby inhibiting the coronavirus pathogenesis pathway (Figure 4B). 

### 2.5. Mapping of Molecules Affected by PA onto the Coronavirus Pathogenesis Pathway

**Figure 5:**
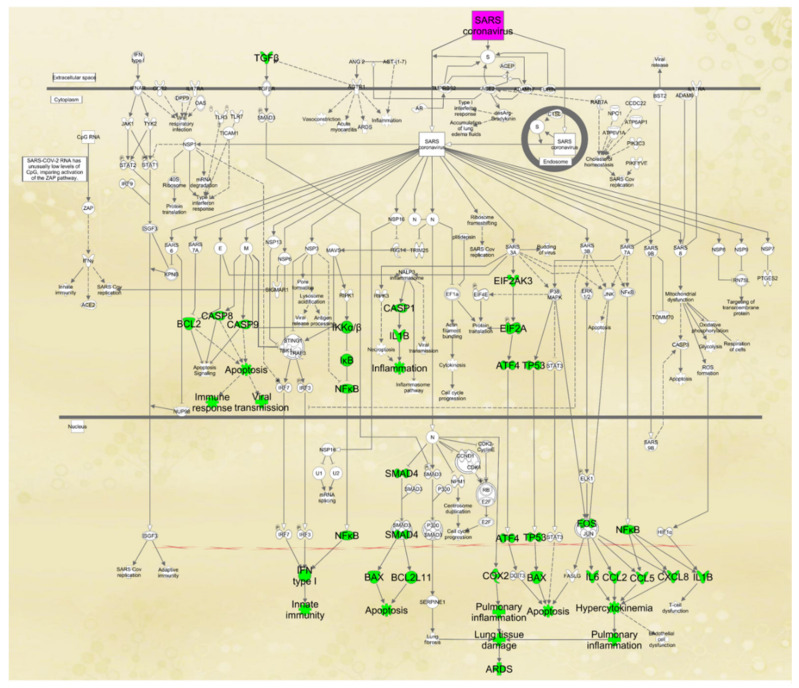
Molecular mediators on the paths from PA to COVID pathogenesis were mapped onto the coronavirus pathogenesis pathway. These mediators and the affected functions were highlighted in green.

In order to examine how the molecules predicted to be involved in the paths from PA to the coronavirus pathogenesis pathway contributed to the development of COVID-19 pathologies, these molecules were mapped onto the coronavirus pathogenesis pathway. An increased level of PA could contribute to apoptosis in three different ways: (1) PA-induced inhibition of BCL2 and activation of BAX, CASP8, and CASP9 induce apoptosis; (2) PA activates EIF2AK3, which, in turn, activates EIF2A. EIF2A then activates the activating transcription factor 4 (ATF4), leading to apoptosis; and (3) PA activates TP53, which in turn activates BAX, leading to apoptosis (Figure 5). Furthermore, an increased level of PA could contribute to inflammation in four different ways: (1) PA-induced activation of IκB kinase (IKK) inactivates IκB, thereby activating NFκB. Activated NFκB induces the expression of type I Interferon which is critical for innate immunity; (2) PA-induced activation of CASP1 enhances the production of IL1β, which enhances inflammation; (3) PA also activates PTGS2 (COX2), which leads to pulmonary inflammation and lung tissue damage; and (4) PA activates FOS and NFκB, which leads to the increased production of IL6, CCL2, CCL5, CXCL8, IL1β, leading to hypercytokinemia and pulmonary inflammation (Figure 5). 

### 2.6. PA Modulation of ACE2

**Figure 6:**
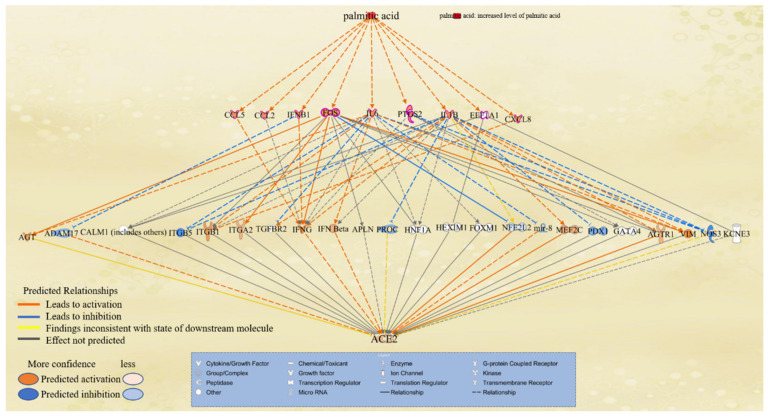
The paths from PA to ACE2 mediated by molecules overlapped by PA- and COVID-19-associated molecules and involved in PA-induced activation of the coronavirus pathogenesis pathway. The paths were explored using the “Path Explorer” tool in IPA, and elevated level of PA was simulated using the “MAP” tool in IPA.

The overlapped molecules between PA- and COVID-19-associated molecules were then compared with the molecules on the paths from PA to the coronavirus pathogenesis pathway. Nine overlapped molecules were found to be involved in PA-induced activation of the coronavirus pathogenesis pathway, namely FOS, PTGS2, IL1B, CXCL8, CCL2, CCL5, IL6, IFNB1, and EEF1A1. The “Path Explorer” tool was then used to investigate the potential paths from PA to ACE2 via these mediators, and then “MAP” tool was used to predict how elevated level of PA might affect the expression of ACE2. Consistently, PA was found to activate all nine molecules, as shown in Figure 6. These nine molecules have differential effects on various mediators, leading to a mild increase in the activity of ACE 2 (Figure 6). 

## 3. Discussion

It is well-recognized that obesity is associated with elevated levels of circulating FFAs [38]. Recently, more severe outcomes have been reported in obese COVID-19 patients [39]. Therefore, this study was undertaken to examine the molecular mechanisms by which FFAs affect the pathogenesis of COVID-19. Among the 35 biological molecules overlapped between PA- and COVID-19-associated molecules retrieved from QKB, 20 were involved in immune regulation, and some of these molecules, such as EEF1A, IL1β, IFNB1, IL6, CCL2, CXCL8, CCL5, PTGS2, and FOS, were mapped to the paths from PA to the coronavirus pathogenesis pathway. We found that PA may (1) activate the transcription factors, such as FOS and NF-κB, that regulate the expression of inflammatory mediators; (2) increase the activity of molecules contributing to apoptosis, such as EIF2AK3, ATF4, CASP8, CASP9, and BAX; (3) enhance the activity of PTGS2; and (4) increase the production of cytokines, including IL1β and IL6, and chemokines, including CCL2, CCL5, and CXCL8, contributing to hypercytokinemia, inflammation, and pulmonary inflammation. These findings suggest that PA may contribute to the worsening of COVID-19 pathologies via immune regulation and apoptosis. We also found that n-3 fatty acids may attenuate inflammation and apoptosis by decreasing the activities of mediators on the paths from PA to the Coronavirus Pathogenesis Pathway, such as IL1β, CCL2, PGTS2, and BAX. Further investigations on how obesity and COVID-19 affect the activities of these key molecules may provide further insight into the molecular mechanisms of augmented COVID-19 pathologies in obese patients. 

Chronic low-grade inflammation is central to the development of various obesity-associated pathologies including insulin resistance, diabetes, cardiovascular diseases, and NAFLD [29]. Our analysis showed that chronic low-grade inflammation in obesity may also augment COVID-19 pathologies. There is accumulating evidence that n-3 fatty acids ameliorate inflammatory reactions, modulate neuroinflammation, reduce oxidative stress, and mitigate coagulopathy [40,41,42,43]. Docosahexaenoic acid (DHA)-flurbiprofen combination ameliorates obesity-associated meta-inflammation in rats fed on a high-carbohydrate high-fat diet [44]. Co-supplementation of omega-3 and vitamin D has remarkable beneficial effects on autism spectrum disorder [45]. n-3 fatty acids are also precursors necessary for the formation of specialized pro-resolving mediators (SPMs) by macrophages and neutrophils [46]. Treatment with n-3 fatty acids increase the levels of SPMs in circulation, including resolvins, protectins, and maresins, which are derived from eicosapentaenoic acid (EPA) and DHA [47]. n-3 fatty acids supplementation increases the survival in mice infected with *Streptococcus pneumoniae*, and decreases overall lung tissue inflammation and cell death [48]. Studies have suggested that n-3 fatty acids together with anti-inflammatory drugs could decrease inflammation and thrombotic complications associated with COVID-19 [46,49]. Consistently, levels of EPA and DHA in red blood cells are inversely associated with risk for death from COVID-19, even though the trend is not statistically significant [50].

Angiotensin-converting enzyme 2 (ACE2), a monocarboxypeptidase, has been identified as the main cell surface receptor for the SARS-CoV-2 spike protein [51]. ACE2 is expressed in wide-range types of cells corresponding to all major human tissues and organs including the lungs, intestines, heart, brain, kidney, liver, muscle, and blood vessels [10,52]. We found that PA may increase the activities of PTGS2, chemokines, such as CCL5, CCL2, and CXCL8, cytokines, such as IFNB1, IL6, and IL1β, transcription factors, such as FOS; and translation regulators, such as EEF1A1, which, in turn, mildly increases the activity of ACE2. Consistently, expression of ACE2 is upregulated in adipocytes of patients with obesity and diabetes as compared to non-obese individuals, which may provide a target for viruses and increase the susceptibility of obese individuals to infection by SARS-CoV-2 [53]. It is worthwhile to examine how ACE2 expression is affected in other tissues of obese subjects and how ACE2 expression level may affect the outcome of COVID-19 pathologies. 

Besides respiratory and gastrointestinal tracts, there is accumulating evidence that SARS-CoV-2 has a tropism for the nervous system, leading to neurological sequelae including immune-mediated demyelinating disease, cerebrovascular damage, and neurodegeneration [54,55,56]. SARS-CoV-2 may infect neurons, microglia, astrocytes, pericytes/endothelial cells, ependymocytes/choroid epithelial cells, and neural stem/progenitor cells [57,58]. SARS-CoV-2 infection of the brainstem may also cause respiratory center dysfunctions [59]. Dysregulation of hormone and neurotransmitter signaling have also been suggested to contribute to the neuropathogenic sequelae of SARS-CoV-2 infection [60]. While the mechanisms by which SARS-CoV-2 impairs the CNS are not completely known, neuroinflammation and blood–brain barrier (BBB) disruption have been suggested to contribute to neurological symptoms and poor prognosis [60,61,62]. Indeed, profound neuroinflammatory changes have been reported in COVID-19 patients [63], which could be due to direct infection of the CNS, microglia activation, and activated peripheral immune response to SARS-CoV-2 infection [56,64], all of which could be worsened by obesity [65,66]. Significant peripheral inflammation during COVID-19 could disrupt the BBB and contribute to COVID-19 neuropathologies [67]. In our study, neuroinflammation was identified as the most significant canonical pathway associated with PA. Studies in animals have shown that metabolic dysfunction from high fat diets leads to increased brain inflammation, reactive gliosis, and decreased brain volume [68]. 

This study used the 408 biological molecules associated with COVID-19 in QKB for our bioinformatic analysis. Among these 408 biological molecules, 181 were associated with severe COVID-19, and 13 were associated with mild COVID-19. Among these 13 molecules, nine were associated with both mild and severe COVID-19 and four were associated with mild COVID-19 only (Table 1). The nine biological molecules associated with both mild and severe COVID-19 included receptors, namely androgen receptor (AR), nuclear receptor subfamily 3 group C member 1 (NR3C1)/glucocorticoid receptor, NR3C2/mineralocorticoid receptor, toll-like receptor (TLR)7 and TLR9; cytokine/chemokines, namely C-C motif chemokine ligand 5 (CCL5); signaling mediators, namely Janus Kinase 1 (JAK1); and enzymes, namely phosphodiesterase 5A (PDE5A) and cytochrome P450 oxidoreductase (POR). The four molecules associated only with mild COVID-19 included cereblon (CRBN), hemoglobin (HBA)1/HBA2, JAK2, and tumor necrosis factor ligand superfamily member 11 (TNFSF11) (Table 1). Moreover, while only one chemokine/cytokine molecule was found to be associated with mild COVID-19, 15 chemokine/cytokine molecules were found to be associated with severe COVID-19 in QKB, which is consistent with the evidence of cytokine storm worsening the prognosis of COVID-19 [69], and the call for immunologic treatments in severe COVID-19 cases but not in mild COVID-19 cases [69]. It would be helpful to identify molecular profiles specific for mild and severe COVID-19, respectively, and use this molecular profile to facilitate the diagnosis and design of therapeutic interventions including immune modulation. 

Several limitations of this study may potentially affect the findings. First, as more progress is made on the understanding of COVID-19 and obesity, more molecules and relationships will be identified, which will improve the comprehensiveness of COVID-19- and PA-associated molecules in QIAGEN’s QKB database for IPA analysis. Secondly, IPA analysis gives all the molecules in the pathway an equal weight without taking into account the varied significance of different molecules or whether the molecule is inherent to the interactions [70]. Finally, considering the importance of elevated levels of free fatty acids (FFAs) in obesogenic pathologies [23,24], this study focused on the analysis of PA and n-3 fatty acids on the pathogenesis of COVID-19. Obesity-associated metabolic disturbances, reactive oxidative stress, endoplasmic reticulum (ER) stress, and pathologies in multiple tissues and organs [23,24,25,71] may further complicate COVID-19 pathogenesis. It would be helpful to examine how obesity-associated metabolic disturbances and pathologies augment COVID-19 pathologies.

In summary, our study investigated the relationship between FFAs and COVID-19 pathogenesis using IPA analysis of available data. We found that PA, a saturated fatty acid, contributes to the pathogenesis of COVID-19 by modulating the molecules involved in immune regulation and apoptosis. Key molecules that contribute to PA augmentation of COVID-19 pathologies included PTGS2; cytokines, such as IL1β, IL6, and IFNB1; chemokines, such as CCL2, CCL5, and CXCL8; translation regulators, such as EEF1A; transcription factors, such as FOS and NFκB; and molecules involved in apoptosis, such as BAX. We found that PA may also modulate the expression of ACE2. Furthermore, n-3 fatty acids may decrease the activity of CCL2, IL1β, BAX, and PTGS2. It should be noted that proinflammatory cytokines such as TNF-α, IL6, and IL1β may promote the disruption of lipid metabolism resulting in systemic inflammation [72], and that COVID-19 itself has been associated with dyslipidemia [46], which may worsen obesity-associated dyslipidemia. These factors may further contribute to COVID-19 complications. Our study should shed light on how PA and n-3 fatty acids could affect the coronavirus pathogenesis pathway by modulating the activity of molecules involved in immune response and apoptosis.

## 4. Materials and Methods

### 4.1. Ingenuity Pathway Analysis (IPA) Software

The license for Ingenuity Pathway Analysis (IPA), a web-based bioinformatics tool, was purchased from QIAGEN (Germantown, MD, USA) [73]. IPA provides insightful data analysis building on comprehensive, manually curated information in the QIAGEN Knowledge Base (QKB). QKB contains over 7 million findings of the granular context and causality that have been curated and captured by QIAGEN scientists from biomedical literature. By combining this expansive knowledge base with statistical analysis and computational modeling, IPA makes it possible to perform causal pathway analysis to identify and group interconnected genes in a network or pathway, and annotate functional changes caused by differences in gene expression.

### 4.2. IPA Analysis

IPA Core Analysis was used to identify the most significant biological canonical pathways that the molecules in the dataset are involved in [70,74]. Statistical analysis was performed using Fisher’s exact test with the Benjamini–Hochberg method of multiple testing correction to assess which biological attributes, such as a pathway or biological function, were significantly associated with the genes in the dataset [75].

Molecules associated with fatty acids and COVID-19 were retrieved from QKB with chemicals trimmed from the dataset. The dataset of molecules associated with fatty acids was expanded using the “Grow” tool in IPA with chemicals trimmed to focus on biological molecules. The “Path Explorer” tool was used to examine the shortest path of relationships between different molecules and pathways. The “Molecule Activity Predictor (MAP)” tool was used to simulate activation or inhibition of a specific molecule and predict the response of downstream molecules in the pathway. Data retrieval and analysis was conducted between 15 November 2020 and 31 March 2021.

## 5. Conclusions

Using Ingenuity Pathway Analysis, our study found that one of the top canonical pathways affected by PA was the coronavirus pathogenesis pathway, mediated by a set of key molecules involved in immune regulation and apoptosis, and that PA may also mediate the activity of ACE2, the main cell surface receptor for SARS-Cov-2 spike protein. In contrast, n-3 fatty acids may have antagonistic effects on some of the key mediators, thereby alleviating COVID-19 pathologies. Our results shed light on how PA and n-3 fatty acids may modulate the coronavirus pathogenesis pathway, and thereby affect COVID-19 pathologies.

## Figures and Tables

**Table 1:** Biological molecules associated with mild and severe COVID-19.

Molecules Associated with Mild COVID-19 Only	Molecules Associated with Both Mild and Severe COVID-19	Molecules Associated with Severe COVID-19 Only
CRBN, HBA1/ HBA2, JAK2, TNFSF11	AR, CCL5, JAK1, NR3C1, NR3C2, PDE5A, POR, TLR7, TLR9	ACSL1, ACTB, ADAR, AHNAK, AHSG, ALB, AP1S2, APCS, APOA1, APOA2, APOD, APOH, APOL1, APOM, ARL4C, C1RL, C4A/C4B, C4BPA, C5, C6, C8A, CALCA, CCL2, CCL3, CCL4, CCL7, CD38, CD3D, CD3G, CD74, CFB, CFHR5, CFI, CFP, CLEC3B, CLU, CPN1, CRP, CSF3 CX3CR1, CXCL10, CXCL8, DUSP1, DYSF, EEF1A1, EEF2, EIF1, EIF2AK2, EIF4B, ETS1, F5, FCGR3A/FCGR3B, FCN2, FGFBP2, FLNA, FOS, GNLY, GPLD1, GZMB, HBB, HLA-DMB, HLA-DPA1, HLA-DPB1, HLA-DQB1, HLA-DRA, HLA-DRB1, HLA-DRB5, HLA-E, HLA-F, HRG, HSPA5, IER2, IFI27, IFI44, IFI44L, IFI6, IFIT3, IFITM3, IFNB1, IFNG, IGHV3-73, IGHV4-28, IGLC3, IL10, IL17A, IL1B, IL1RN, IL27, IL32, IL6, IL6R, IL7, IL9, IRF7, ISG15, ITIH3, ITIH4, JUNB, LGALS1, LGALS3BP, LOC100132215, MMRN1, MX1, MX2, MYOM2, OAS2, OAS3, ORM1, ORM2, PABPC1, PARP14, PARP9, PCYOX1, PF4, PFN1, PIM1, PKM, PLAC8, PLBD1, PLEK, PPBP, PPIA, RACK1, RBP4, RGS2, RNA28S5, RNASE2, RSAD2, RSRP1, S100A12, S100A8, S100A9, SAA1, SAA2, SAA4, SAMD9, SAMD9L, SELL, SERPINA3, SIGLEC1, SLC25A6, SP100, SPARC, STAT1, SYNE1, TAGLN2, TNF, TNFAIP2, TPT1, TRGC1, TRGC2, TRIM22, TUBA1A, TUBA1C, TUBA3C/TUBA3D, TUBA4A, TUBA8, TUBB1, TUBB2A, TUBB3, TUBB4A, TUBB4B, TUBD1, TUBE1, TUBG1, TUBG2, TXNIP, VCAM1, VCAN, VTN, XAF1, ZFP36L2

## Data Availability

The biological molecule datasets used in this study were retrieved from QIAGEN Knowledge Base within Ingenuity Pathway Analysis (IPA) (https://www.qiagen.com/qiagen-ipa (accessed during 15 November 2020 and 31 March 2021)) to allow exploration in the context of other datasets. IPA is commercially available from QIAGEN.
